# Intraoperative Diagnostic Methods for Superior Mesenteric Artery Stenting in Chronic Mesenteric Ischemia

**DOI:** 10.3390/jcm14238507

**Published:** 2025-11-30

**Authors:** Alexandra A. Brandtzäg, Jonas P. Eiberg, Mikkel Taudorf, Lars Lonn, Timothy A. Resch

**Affiliations:** 1 Department of Vascular Surgery, The Heart Center, Copenhagen University Hospital-Rigshospitalet, 2100 Copenhagen, Denmark; alexandra.annelie.brandtzaeg@regionh.dk (A.A.B.);; 2 Department of Clinical Medicine, Faculty of Health and Medical Sciences, University of Copenhagen, 2200 Copenhagen, Denmark; 3 Copenhagen Academy for Medical Education and Simulation (CAMES), Centre for HR & Education, The Capital Region of Denmark, 2100 Copenhagen, Denmark; 4 Department of Radiology, Copenhagen University Hospital-Rigshospitalet, 2100 Copenhagen, Denmark

**Keywords:** superior mesenteric artery, endovascular procedure, digital subtraction angiography, intra-arterial pressure measurement, cone beam computer tomography

## Abstract

**Background/Objectives:** Chronic mesenteric ischemia (CMI) due to superior mesenteric artery (SMA) stenosis can be effectively treated with endovascular therapy (EVT). Appropriate intraoperative assessment is crucial for ensuring technical success and long-term patency. This study assesses intra-arterial pressure measurement (IAPM), and cone beam computed tomography (CBCT) for detecting residual stenosis during SMA stenting in CMI. **Methods:** This prospective study included 50 consecutive elective patients with symptomatic, significant SMA stenosis scheduled for EVT. The patients in this study were a subset of an ongoing randomized trial with a different primary objective. Intraoperative diagnostic tools—digital subtraction angiography (DSA), IAPM and CBCT were performed after stent placement. Technical success was defined as <30% residual stenosis on DSA, a residual pressure gradient of <10 mmHg with IAPM and full stent expansion on CBCT. **Results:** Although there was a fair agreement between DSA and CBCT (Kappa 0.294, *p* = 0.024), the Odds ratio suggests that DSA detected fewer positive cases compared to CBCT (Odds ratio 0.176; 95% CI: 0.004–1.37; *p* = 0.13). DSA also differed significantly from IAPM (Kappa = 0.016, *p* = 0.882), (Odds ratio = 0.167; 95% CI: 0.018–0.749; *p* = 0.013), suggesting DSA under-detects residual stenosis (>10 mmHg). No significant difference was found between CBCT and IAPM (Kappa = 0.201, *p* = 0.161) (Odds ratio: 2.25, 95% CI: 0.628–10.0%; *p* = 0.27) indicating comparable results. **Conclusions:** DSA overlooks clinically important residual stenosis that could be treated during the primary procedure. CBCT aids structural assessment of the stent and allows for on-table optimizing of the procedural outcomes.

## 1. Introduction

Endovascular therapy (EVT) of the superior mesenteric artery (SMA) is the preferred treatment in suitable cases of chronic mesenteric ischemia (CMI) [1]. The most common cause of CMI is stenosis of the SMA caused by atherosclerosis, accounting for approximately 90% of cases [2]. However, during the first year after SMA stenting, a high rate of restenosis and relapsing symptoms is observed with a higher rate in women and in vessel diameters < 6 mm [2,3].

Restenosis affects up to 40% of stented patients, with one-third to half requiring reintervention [2,4]. In a national, retrospective cohort, approximately 7% of patients received reintervention due to symptomatic in stent restenosis of the SMA within one year after the primary procedure [5]. The high recurrence rate within the first year as well as the seemingly high secondary patency after reintervention suggests that intraoperative undetected technical failures are common. Digital subtraction angiography (DSA) remains the main intraoperative modality when evaluating stent placement during endovascular procedure. However, the large amount of symptomatic and asymptomatic restenosis suggests that DSA may not reliably detect all residual stenosis during the primary surgery.

During the procedure, it is often difficult to evaluate the success of the stenting consistently, as it depends on the visual interpretation by the operator, often utilizing angiography as the sole modality when evaluating technical outcome [6]. Tallarita et al. demonstrated that the location of restenosis coincided with technical flaws, including missed lesions and partial stent compression, flaws that increased the risk of postprocedural restenosis of >30% [7].

Intra-arterial pressure measurement (IAPM) enables direct assessment of trans-stenotic pressure gradients and is considered by many to be the gold standard for evaluating the functional significance of arterial stenoses, as it is based on physiological rather than anatomical criteria [4,8,9]. Despite this, IAPM is not routinely implemented across centers performing renovisceral endovascular procedures and is primarily reserved for cases where the significance of the stenosis is uncertain [10].

IAPM is a common and widely described procedure during coronary angiography, with dedicated pressure wires and standardized protocols [11,12]. In peripheral arterial disease (PAD), IAPM before and after vasodilator administration and calculation of Fractional Flow Reserve, is predictive of patency after revascularization [6].

CBCT (cone beam computer tomography) is increasingly used during complex endovascular aortic procedures, to assess the structural integrity of the renovisceral target vessel stents. Compared with DSA alone, CBCT more effectively detects stent compression and kinking [13,14].

Whereas previous guidelines define successful revascularization as <10 mmHg gradient with IAPM and <30% reduction in diameter on DSA, newly published mesenteric guidelines do not mention specifically what determines technical success [1,15]. The European Society for Vascular Surgery (ESVS) guidelines refer to a meta-analysis where most studies defined technical success as a residual diameter reduction of <30% on completion angiogram and a residual pressure gradient between 5 and 10 mmHg [16]. They also specifically quote the current lack of reporting standards as an important focus of future research.

This study aims to explore IAPM and CBCT as additional intraoperative diagnostic methods after SMA stenting for CMI, with a specific focus on their ability to detect residual stenosis compared to standard DSA.

## 2. Materials and Methods

### 2.1. Study Design

**Figure 1:**
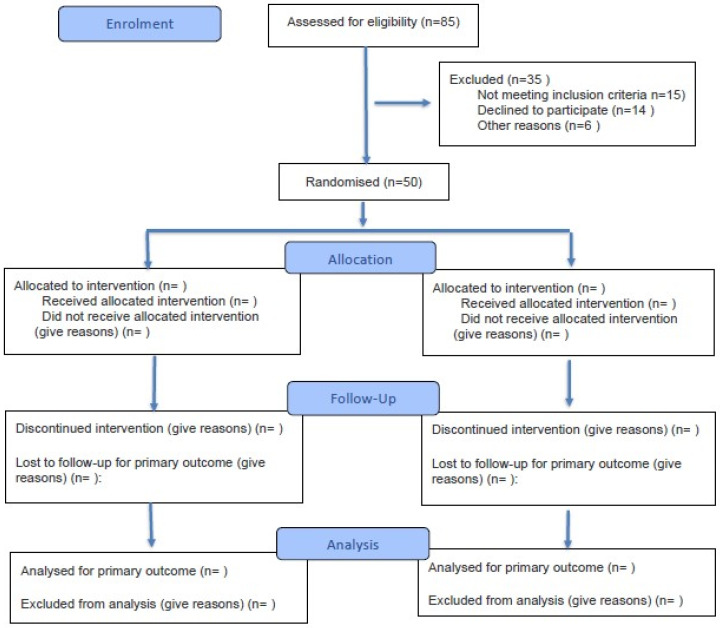
CONSORT Flow Diagram. Modified flow diagram of the progress through the phases of a randomised trial of two groups (including enrolment). Adapted from Hopewell et al. [18].

This is a sub-analysis, based on prospectively collected data in the COMESS trial. The patients included represent a subset of an ongoing randomized trial of covered vs. bare metal stents for treatment of SMA stenosis (COMESS trial, Clinical trials.org # NCT05244629) [17]. Data was collected prospectively. Because the COMESS trial is ongoing, the CONSORT flow diagram (Figure 1) reflects the participants included in this sub-analysis.

### 2.2. Patients

In addition to relevant symptoms of CMI, included patients had a de novo SMA stenosis of >50% on CTA with atherosclerotic or atherothrombotic etiology. Patients were not included if they had signs of acute mesenteric ischemia (AMI) or if they had previously been treated in the SMA. Patients were treated with either a bare metal stent (BMS) (BeSmooth, Bentley Innomed GmBH, Hechingen, Germany) or a stent graft (BeGraft, Bentley Innomed GmBH, Hechingen, Germany). Both stents contain a cobalt chrome base and are identical in structure. The covered stent (CS) has a polytetrafluorethylene (PTFE) covering [17].

### 2.3. Digital Subtraction Angiography

All procedures were performed in an Angio suite with a ceiling mounted Philips c-arm (Azurion 7 M20 Philips systems 2023 Nederland B.V. Koninklije). During the procedure, DSA is performed before and after stent/stent graft placement in an antero-posterior and lateral projection [5,19]. During the non-selective aortic angiography 15–20 mL contrast (Visipaque 270 mg/mL; GE Health Care, Princeton, NJ, USA) is administered allowing visualization of the mesenteric vessels [19]. A <30% residual stenosis on DSA after stenting was considered technical success [15].

Residual stenosis on DSA was measured using the following algorithm: ((B − A)/B) × 100, where A is the minimal luminal diameter within the stent, and B the largest nominal luminal diameter just distal to the stent, in the lateral projection [4,20].

### 2.4. Intra-Arterial Pressure Measurement

Intra-arterial pressure measurement was performed with a dedicated pressure wire, Omniwire (Koninklijke Philips N.V., Amsterdam, The Netherlands), measuring the MAP simultaneously in the aorta and the SMA. IAPM is measured 1–2 cm distal from the stenosis performed without vasodilators [21]. The Omniwire is calibrated in the aorta, prior to advancing it across the SMA stenosis. After the lesion is crossed, the IAPM is recorded simultaneously in the aorta and the SMA for 15 s. The average MAP gradient between the aorta and SMA is then calculated. The trans-stenotic pressure measurement is repeated after stent placement in the SMA [12,22]. A residual pressure gradient of <10 mmHg after stenting was considered technical success [15].

### 2.5. Cone Beam CT

Non-contrast enhanced cone beam CT (nCBCT)—(Azurion 7 M20 Philips systems 2023 Nederland B.V. Koninklije) was performed as a final step during the procedure, to assess the integrity or compression of the stent. CBCT was performed using a standard abdominal protocol. The CBCT acquisition had a duration of 5 s and produced 313 images and was performed without contrast. All CBCT scans followed the same protocol, ensuring similar exposure. CBCT is performed without contrast as the resolution is too low to detect intraluminal defects even when using contrast enhancement. The inner diameter at the narrowest part of the stent is noted, in addition to any structural abnormalities of the stent. Technical success according to CBCT was considered if the was stent fully expanded to nominal diameter. All procedures were performed by experienced interventional radiologists.

### 2.6. Data Analysis and Statistics

A Fleiss kappa analysis was performed to see the overall agreement between the modalities. To illustrate the pairwise agreement between the DSA, IAPM and CBCT a Cohen’s Kappa analysis was performed. The strength of agreement based on the Cohens kappa coefficient was then interpreted according to Landis and Koch, as follows: <0.00 poor, 0.00–0.20 slight, 0.21–0.40 fair, 0.41–0.60 moderate, 0.61–0.80 substantial, 0.81–1.00 almost perfect. A Mcnemar test was performed to assess the differences in outcomes between the diagnostic modalities. In addition, 2 × 2 contingency tables were constructed to illustrate how patients were categorized based on technical results. Furthermore, the sensitivity and specificity were estimated, computing the accuracy, sensitivity and specificity, positive predictive value (PPV) and negative predictive value (NPV). IAPM was used as the reference standard for sensitivity and specificity. All DSA stenosis measurements were performed perioperatively by one person (AB) to ensure consistency, thus no inter-observer variability analysis was deemed necessary. All statistical analysis were performed in R (v.4.2.2), and a *p*-value of <0.05 was considered statistically significant.

### 2.7. Ethics

The trial is in accordance with the Declaration of Helsinki. Written informed consent was obtained from patients for the Copenhagen Mesenteric Stent Study (COMESS) study which was approved by the Regional Ethics Committee in the Capital Region of Denmark, H-22004003.

## 3. Results

Fifty patients with CMI and SMA stenting were included (Table 1). IAPM and CBCT were performed after DSA to evaluate the trans-stenotic pressure gradient and the structural integrity of the stent. IAPM was performed in all patients. CBCT was not possible to perform in six cases due to technical issues yielding a total of 44 paired measurements, which was used in the analysis. The mean post-stent pressure gradient was 8.64 ± 9.03 mmHg, and the mean residual stenosis was 11.83 ± 10.38% on DSA (Table 2).

### 3.1. Comparison of All Modalities After Stent Placement

Agreement between DSA, IAPM and CBCT was slight (Kappa = 0.134, *p* = 0.123) and not statistically significant, suggesting that the modalities cannot be used interchangeably.

### 3.2. Comparison Between DSA and IAPM After Stenting

The agreement between DSA and IAPM was minimal (Kappa = 0.016, *p* = 0.882), and not statistically significant. There was a statistically significant difference between DSA and IAPM, (Odds ratio = 0.167; 95% CI: 0.018–0.749; *p* = 0.013), suggesting that DSA under-detects cases with a pressure gradient of >10 mmHg (Table 3). DSA is sensitive at detecting cases of <10 mmHg, with a sensitivity of 93.5% (*p* < 0.001; 95% CI: 78.6–99.2). However, DSA has a low specificity of 7.7% (95% CI: 0.2–36.0%; *p* = 0.003) and does not detect all cases >10 mmHg. The PPV of DSA was 70.7% (95% CI: 54.5–83.9%; *p* = 0.0115), showing a moderate diagnostic value whereas the NPV was 33.3% (95%CI 0.84–90.6; *p* = 1.00).

### 3.3. Comparison Between DSA and CBCT After Stenting

**Figure 2:**
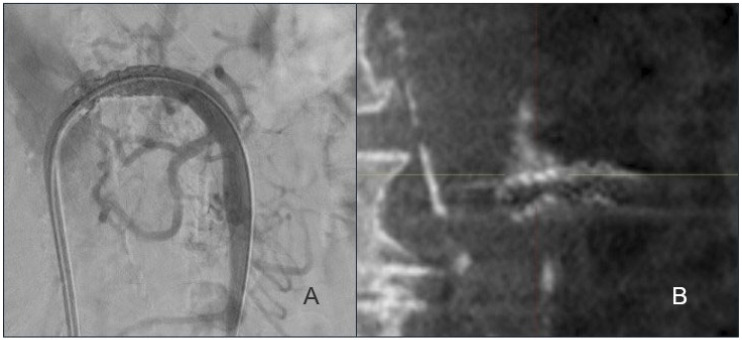
Completion imaging after SMA stent placement. Completion angiography (DSA) following stent placement (**A**). Stent placement visualized on CBCT (**B**).

Although a fair agreement was observed between DSA and CBCT (Kappa 0.2944, *p* = 0.024), the Odds ratio suggests that DSA detected fewer positive cases compared to CBCT, which did not reach statistical significance (Odds ratio 0.176; 95% CI: 0.004–1.37; *p* = 0.13) (Figure 2). In cases where the stent appears fully expanded on CBCT, DSA demonstrated a sensitivity of 97.2% (95% CI 85.5–99.9%; *p* < 0.001) (Table 4). However, the specificity was 25% (95% CI 3.2–65.1%; *p* = 0.29) suggesting that DSA may not correctly identify structural stent problems seen on CBCT. DSA demonstrated a PPV of 81.8% (95% CI 67.3–91.8%; *p* < 0.001) for detecting findings confirmed on CBCT, while the NPV was 21.4% (95% CI 4.7–50.8; *p* = 0.057).

### 3.4. Comparison of CBCT with IAPM

Agreement between the CBCT and IAPM (Kappa = 0.201, *p* = 0.161) was slight and did not reach statistical significance. No significant difference was found between CBCT and IAPM (Odds ratio: 2.25, 95% CI: 0.628–10.0%; *p* = 0.27) suggesting CBCT and IAPM performed similarly. CBCT had a moderate to high sensitivity of 75.0% (95% CI: 57.8–87.9%; *p* = 0.0039) when determining positive and negative cases (Table 5). There was a low specificity 50% (95% CI: 15.7–84.3%, *p* = 1.00) when identifying negative cases, indicating that CBCT may be prone to “false” positive cases, however this did not reach statistical significance. CBCT demonstrated a PPV of 87.1% (95% CI: 70.2–96.4%; *p* < 0.001), when detecting findings confirmed on IAPM, while the NPV 30.8% (95% CI 9.1–61.4%; *p* = 0.267).

## 4. Discussion

Our findings indicate that DSA, IAPM, and CBCT are not interchangeable but rather complementary diagnostic methods. Although performing all three modalities during EVT may not always be feasible due to added procedural time and cost, reliance on DSA alone appears insufficient based on our results and the prior literature.

The most recent guidelines do not define intraoperative imaging protocols for assessing stent integrity or technical success [1]. Current intraoperative evaluation remains largely based on established criteria of a <10 mmHg pressure gradient and <30% residual stenosis on completion DSA [15]. During standard procedures, DSA remains the primary modality for assessing stent outcomes, with limited use of additional diagnostic techniques. However, both CBCT and IAPM are well-established in other endovascular fields to confirm technical success [12,23].

DSA provides valuable flow assessment and can identify technically successful cases, but it lacks specificity compared with IAPM and CBCT. It fails to capture structural abnormalities that may influence procedural outcomes. Tallarita et al. demonstrated that restenosis frequently occurred at sites of technical imperfections, such as missed lesions or partial stent compression, which were associated with residual stenosis exceeding 30% [7]. Our findings similarly suggest that DSA may overlook stent-related structural problems detected by CBCT and underestimate cases with residual pressure gradients >10 mmHg.

Both CS and BMS were used in this study for the treatment of SMA stenosis. Both are made of a cobalt chrome base and are identical in structure, reducing the risk of potential stent-related artifact. The only difference is the PTFE covering in the CS; however, this is unlikely to generate major visual difference on CBCT, seeing as CS and BMS have identical frameworks.

CBCT and IAPM performed comparably, both outperforming DSA in identifying cases lacking technical success. CBCT, however, appears more prone to false positives; in other words, CBCT may over-detect structural changes that do not correspond to relevant defects. While IAPM provides a functional assessment of post-stenting hemodynamics, it may be influenced by distal disease or collateral circulation. Nevertheless, when used together with DSA, IAPM can localize the segment with the most significant pressure gradient.

A retrospective study demonstrated that intraoperative CBCT detected clinically relevant structural defects, allowing immediate correction in two-thirds of cases [24]. The same study reported that the current 5 s acquisition protocol delivered a radiation dose of approximately 42 Gy·cm^2^ (IQR 33–47 Gy·cm^2^). CBCT, even without contrast, provides detailed structural information within a short acquisition time. In our analysis, CBCT demonstrated low specificity, suggesting potential false positives in identifying residual stenosis, though this did not reach statistical significance.

Given these findings, inclusion of an adjunctive diagnostic modality in the intraoperative workflow appears warranted. Performing all three methods, however, may be impractical in routine clinical settings as it increases the procedural time, radiation dose, as well as cost of the procedure. There was no significant difference in performance observed between IAPM and CBCT. CBCT effectively identified cases requiring further intervention and may serve as a suitable complement to DSA. The combined use of DSA and CBCT could potentially obviate the routine need for IAPM, reserving IAPM for uncertain cases to confirm findings that require further intervention.

### Limitations

CBCT was not performed in six cases, due to technical malfunction. The use of additional diagnostic methods, including IAPM, increased both procedural time and cost. This single center study did not assess the correlation between long-term postoperative symptom relief and the diagnostic methods, as its primary focus is to evaluate potential residual stenosis and the ability of additional diagnostic methods to detect it. Future studies are needed to assess the correlation between intraoperative findings and symptom relief to evaluate the effect of potential intraoperative technical flaws.

## 5. Conclusions

Based on the results in this study an additional diagnostic modality may be considered as an adjunctive intraoperative tool pending further validation, to evaluate the technical outcome after SMA stenting. DSA seems to underestimate residual stenosis that could be treated during the primary procedure. In addition to DSA, CBCT or IAPM may be added to the diagnostic procedure, to aid in structural assessment of the stent and optimizing procedural outcomes.

## Figures and Tables

**Table 1:** Overview of patient demographics.

Age	73 ± 8.1 Years
**Gender (female)**	56% (28)
**BMI**	23.2 kg/m^2^ ± 5.4 kg/m^2^
**Smoking status:**	
**Current**	30% (15)
**Former**	54% (27)
**Never**	16% (8)
**Comorbidities:**	
**Diabetes Mellitus**	26% (13)
**Cardiac**	20% (10)
**COPD**	24% (12)
**Hypertension**	70% (35)
**Peripheral vascular disease**	18% (9)

**Table 2:** Description of lesion on DSA.

Mean Stenosis-SMA	73% ± 16.8%
**Residual stenosis-SMA**	11.83% ± 10.38%
**Vessels affected:**	
**One vessel disease (SMA)**	48% (24)
**Two vessel disease (CA and SMA)**	44% (22)
**Two vessel disease (SMA and IMA)**	0% (0)
**Three vessel disease (CA, SMA, IMA)**	8% (4)
**Morphology of stenosis–SMA: **	
**Simple stenosis**	90% (45)
**Complex stenosis**	10% (5)
**Placement of stenosis-SMA:**	
**Ostial stenosis**	78% (39)
**Non-ostial stenosis**	24% (12)
**Average diameter-SMA**	6.75 mm ± 0.95 mm

Description of type, placement and morphology of lesion visualized on DSA. Simple stenosis: Uniform, smooth narrowing of the vessel. Complex stenosis: Irregular, tortuous, eccentric narrowing with severe calcifications or involvement of adjacent structures.

**Table 3:** 2 × 2 Contingency table for the comparison between DSA and IAPM.

	IAPM < 10 mmHg	IAPM > 10 mmHg
**DSA > 30% stenosis**	2.3% (1)	4.5% (2)
**DSA < 30% stenosis**	27.3% (12)	65.9% (29)

**Table 4:** 2 × 2 Contingency table for the comparison between DSA and CBCT.

	CBCT-Stent Not Fully Expanded	CBCT-Stent Fully Expanded
**DSA > 30% stenosis**	4.5% (2)	2.3% (1)
**DSA < 30% stenosis**	13.6% (6)	79.5% (35)

**Table 5:** 2 × 2 Contingency table for the comparison between CBCT and IAPM.

	IAPM < 10 mmHg	IAPM > 10 mmHg
**CBCT Stent not fully expanded**	9.1% (4)	9.1% (4)
**CBCT Stent fully expanded**	20.5% (9)	61.4% (27)

## Data Availability

The datasets presented in this article are not readily available because the data are part of an ongoing study. Requests to access the datasets should be directed to the corresponding author.

## Abbreviations

The following abbreviations are used in this manuscript:

EVT	Endovascular therapy
SMA	Superior mesenteric artery
CMI	Chronic mesenteric ischemia
IAPM	Intra-arterial pressure measurement
CBCT	Cone beam computer tomography
nCBCT	Non-contrast enhanced cone beam computer tomography
DSA	Digital subtraction angiography
PAD	Peripheral arterial disease
PPV	Positive predictive value
NPV	Negative predictive value
BMI	Body mass index
CI	Confidence interval
CS	Covered stent
BMS	Bare metal stent
